# Structure–function relationships of the competence lipoprotein ComL and SSB in meningococcal transformation

**DOI:** 10.1099/mic.0.046896-0

**Published:** 2011-05

**Authors:** Afsaneh V. Benam, Emma Lång, Kristian Alfsnes, Burkhard Fleckenstein, Alexander D. Rowe, Eirik Hovland, Ole Herman Ambur, Stephan A. Frye, Tone Tønjum

**Affiliations:** 1 Centre for Molecular Biology and Neuroscience, Institute of Microbiology, University of Oslo, NO-0027 Oslo, Norway; 2 Centre for Molecular Biology and Neuroscience, Institute of Microbiology, Oslo University Hospital (Rikshospitalet), NO-0027 Oslo, Norway; 3 Centre for Immune Regulation, Institute of Immunology, University of Oslo, NO-0027 Oslo, Norway

## Abstract

*Neisseria meningitidis*, the meningococcus, is naturally competent for transformation throughout its growth cycle. The uptake of exogenous DNA into the meningococcus cell during transformation is a multi-step process. Beyond the requirement for type IV pilus expression for efficient transformation, little is known about the neisserial proteins involved in DNA binding, uptake and genome integration. This study aimed to identify and characterize neisserial DNA binding proteins in order to further elucidate the multi-factorial transformation machinery. The meningococcus inner membrane and soluble cell fractions were searched for DNA binding components by employing 1D and 2D gel electrophoresis approaches in combination with a solid-phase overlay assay with DNA substrates. Proteins that bound DNA were identified by MS analysis. In the membrane fraction, multiple components bound DNA, including the neisserial competence lipoprotein ComL. In the soluble fraction, the meningococcus orthologue of the single-stranded DNA binding protein SSB was predominant. The DNA binding activity of the recombinant ComL and SSB proteins purified to homogeneity was verified by electromobility shift assay, and the ComL–DNA interaction was shown to be Mg^2+^-dependent. In 3D models of the meningococcus ComL and SSB predicted structures, potential DNA binding sites were suggested. ComL was found to co-purify with the outer membrane, directly interacting with the secretin PilQ. The combined use of 1D/2D solid-phase overlay assays with MS analysis was a useful strategy for identifying DNA binding components. The ComL DNA binding properties and outer membrane localization suggest that this lipoprotein plays a direct role in neisserial transformation, while neisserial SSB is a DNA binding protein that contributes to the terminal part of the transformation process.

## Introduction

*Neisseria meningitidis*, or the meningococcus, is a common inhabitant of the mucosal surface of the oro- and nasopharynx in humans. The primary concern regarding meningococcus colonization is the sudden occurrence of systemic meningococcal disease that can occur in previously healthy individuals (Stephens *et al.*, 2007). The mechanisms that allow some meningococcus strains to disseminate from their local oro-pharyngeal niche and cause acute systemic disease are poorly understood. Most cases of meningococcal disease are caused by clonal complexes of related sequence types (STs), the so-called hyperinvasive lineages (Yazdankhah *et al.*, 2004). These lineages are underrepresented in healthy carriers, and significant numbers of individuals are colonized with carriage isolates belonging to a set of STs that rarely cause disease (Caugant, 2008). Meningococcus cells exhibit abundant antigenic diversity due to frequent recombination, random mutational events, phase variation and high frequencies of horizontal gene transfer (Davidsen & Tønjum, 2006). Natural transformation is the predominant route for exchange of chromosomal DNA between neisserial strains. Unlike other Gram-negative bacteria, meningococcus is naturally competent for transformation throughout its growth cycle (Jyssum & Lie, 1965). Transformation in meningococcus is a multi-factorial process that requires the presence of the 12 bp DNA uptake sequence (DUS) in the exogenous DNA (Ambur *et al.*, 2007; Goodman & Scocca, 1988), in addition to type IV pilus expression (Frøholm *et al.*, 1973) and RecA-dependent homologous recombination (Koomey & Falkow, 1987). Transformation is coupled to the expression of type IV pili in a number of Gram-negative bacteria (Averhoff, 2004; Swanson *et al.*, 1971; Tønjum & Koomey, 1997). In addition to their role in competence, type IV pili also play a role in adherence (Swanson *et al.*, 1971), twitching motility (Bradley, 1980) and virulence (Caugant, 2008).

Thus, neisserial competence for transformation is dependent on the expression of several pilus-related components, including a number of pilus biogenesis components (Tønjum & Koomey, 1997) and the minor pilin ComP (Wolfgang *et al.*, 1999). The secretin PilQ is associated with translocation of the pilus fibre across the outer membrane by mediating type IV pilus extrusion and retraction (Tønjum *et al.*, 1998). Neisserial PilQ mutants are non-piliated and non-competent for transformation, and PilQ has previously been shown to bind DNA (Assalkhou *et al.*, 2007). Other components suggested to be involved in neisserial transformation include the competence factors ComE (Chen & Gotschlich, 2001), ComA (Facius & Meyer, 1993) and ComL (Fussenegger *et al.*, 1996, 1997). However, all of the DNA binding components involved in the machineries which drive meningococcus transformation and other forms of horizontal gene transfer have not yet been identified.

In order to identify and characterize meningococcus proteins that directly bind DNA, we have previously employed cellular fractionation and a solid-phase overlay assay in the form of South-Western analysis in combination with MS analysis (Lång *et al.*, 2009). In general, the procedure employed proved to be a useful strategy for identifying DNA binding components. Here, the resolution of the solid-phase overlay assay was improved by using 2D gel electrophoresis. In the meningococcus membrane and soluble fraction, respectively, the competence lipoprotein ComL and the neisserial orthologue of the single-stranded DNA binding protein SSB were predominant. The observed DNA binding activity of ComL and SSB was verified by electromobility shift analysis. We propose 3D models for the structures of meningococcus ComL and SSB and define their protein-interacting counterparts. Thereby, meningococcus DNA dynamics relevant for horizontal gene transfer and recombination are further elucidated.

## Methods

### 

#### Bacterial strains, plasmids and growth conditions.

Bacterial strains and plasmids employed in this study are listed in Supplementary Table S1 (available with the online version of this paper). Neisserial strains were grown on blood agar plates in a 5 % CO_2_ atmosphere at 37 °C for approximately 18 h, while *Escherichia coli* strains were cultivated on Luria–Bertani (LB) plates at 37 °C. Selective antibiotics were added when required.

### Cellular fractionation

#### 

##### Enrichment of inner membranes.

Isolation of inner membrane proteins from *N. meningitidis* strains and *Neisseria gonorrhoeae* (the gonococcus) strain N400 has previously been described (Lång *et al.*, 2009). Representative fractions were tested for d-lactate dehydrogenase (LDH) activity as a positive control for the enrichment of inner membrane protein (Balasingham *et al.*, 2007).

##### Enrichment of the soluble fraction.

Neisserial cells were resuspended in 1.5 ml PBS, making a bacterial suspension of OD_600_ 0.7. The cells were pelleted at 13 000 ***g*** for 10 min, resuspended in 20 µl chloroform and incubated at room temperature for 15 min (Ames *et al.*, 1984; Judd & Porcella, 1993). A 0.1 ml volume of 10 mM Tris/HCl, pH 8.0, was added and the preparation was mixed by vortexing and pelleted by centrifugation at 13 000 ***g*** for 20 min at 4 °C. The supernatant containing the soluble fraction was stored at −70 °C.

#### 1D and 2D protein separation and membrane transfer.

1D SDS-PAGE was performed using 10–12 % NuPAGE precast gels and sample buffer according to the manufacturer’s protocol (Invitrogen), and 2D electrophoresis was performed with Immobiline Drystrips and 10–12 % gels according to the manufacturer’s recommendations with slight modifications (Amersham GE Healthcare). In brief, iso-electric focusing (IEF) was performed with nonlinear Immobiline DryStrip gel strips of pH 3–10. Prior to analysis, the proteins were solubilized in rehydration buffer [8 M urea, 2 M thiourea, 4 % CHAPS, 0.5 % Triton X-100, 0.2 % DTT, 2 % IPG buffer (Amersham GE Healthcare)] overnight before being exposed to ultrasound (Branson 3510) for 10 min. In both 1D and 2D SDS-PAGE, proteins were detected by Coomassie blue staining. The solid-phase overlay experiments, with parallel SDS-PAGE and subsequent MS analysis, were repeated at least three times.

#### Solid-phase overlay assay for protein–DNA interaction.

Protein samples and recombinant full-length ComL were screened for DNA binding activity in a solid-phase overlay assay in the form of a South-Western analysis, which has been described previously (Lång *et al.*, 2009). Protein–DNA interactions were assessed by probing the nitrocellulose membranes (Hybond-C Extra, Amersham GE Healthcare) with biotinylated DNA substrates, with or without DUS (see Supplementary Table S2, available with the online version of this paper). Purified DNA glycosylases Fpg (Tibballs *et al.*, 2009) and MutY, *Taq* polymerase (Sigma) and SSB (Sigma) were used as positive controls, while BSA (Sigma) was used as a negative control.

#### Protein identification by peptide mass fingerprinting/MALDI-TOF-MS.

The DNA binding components detected in the solid-phase overlay assay were identified by MS analysis according to previously described methods (Fleckenstein *et al.*, 2004). For presentation of the results, guidelines provided by Molecular and Cellular Proteomics (http://www.mcponline.org/site/misc/ParisReport_Final.xhtml) were taken into account. In brief, tryptic peptides obtained from in-gel digestion were desalted and concentrated using C18 Empore Extraction Disks (Applied Biosystems) placed in GELoader tips (Eppendorf). The retained peptides were eluted onto a stainless steel target plate (Bruker Daltonics) with a solution containing 70 % acetonitrile, 0.1 % trifluoroacetic acid and 10 mg α-cyano-4-hydroxycinnamic acid ml^−1^. After crystallization, the samples were analysed on an Ultraflex II MALDI-TOF/TOF mass spectrometer (Bruker Daltonics) operated in the positive reflector mode. FlexControl v. 2.4 was used for data acquisition, and FlexAnalysis v. 2.2 and BioTools v. 2.2 were used for data evaluation. All MALDI-TOF mass spectra were searched by the web version of Mascot (Matrix Science) against the NCBI, MSDB and Swiss-Prot databases using the following search parameters: one missed cleavage site; fixed modification, carbamidomethylation of cysteine; variable modifications, oxidation of methionine, pyro-glutamate formation of N-terminal glutamine residues; selected taxonomy, *Neisseria meningitidis*; protease, trypsin; peptide mass tolerance ± 0.08 Da.

#### Bioinformatic analyses and search for signature motifs.

The database Uniprot (Boeckmann *et al.*, 2003) provided information on the properties predicted for the DNA binding components identified, whereas disopred2 (Ward *et al.*, 2004) and vsl1 (Obradovic *et al.*, 2005) were employed to assess intrinsic disorder. Searches for functional domains or signature motifs were carried out on the deduced amino acid sequences of meningococcus ComL (AAF41120) and SSB (NP_274471) using the dolop (Babu *et al.*, 2006), prosite (Hulo *et al.*, 2004) and Pfam databases (Bateman *et al.*, 2004). Based on the ComL and SSB sequences, primary prediction of their secondary structures was performed by using the JPred service (Cuff *et al.*, 1998), revealing a prevalence of alpha-helical elements in ComL, while the presence and location of the transmembrane helices were predicted by memsat3 (Jones *et al.*, 1994). The presence of recognized DNA binding motifs was assessed by using the protscale tool (Gasteiger *et al.*, 2005), while the electrostatic charge was calculated by using the charge program from the emboss package (Rice *et al.*, 2000). In the search for structure-dependent DNA binding motifs, the ComL and SSB sequences were first submitted to the DP-Bind server (Hwang *et al.*, 2007) for prediction of sequence-based DNA binding and subsequently to the phyre (Protein Homology/analogY Recognition Engine) server (Bennett-Lovsey *et al.*, 2008) for sequence-based fold recognition and model generation by use of a threading method. Additionally, a homology model for the SSB structure was generated using a swiss-model (Arnold *et al.*, 2006). Rendered images of 3D structures were generated using PyMOL (DeLano, 2002) and Python Molecular Viewer (Sanner, 1999).

#### Mutant construction and phenotypic analysis.

Meningococcus null mutants corresponding to the DNA binding components identified were constructed using the same strains, plasmids and methods described previously (Supplementary Table S1; Lång *et al.*, 2009) and tested with regard to natural competence for transformation and other pilus-related phenotypes, such as colony morphology and the number of pili. The PCR primers employed in the construction of mutants are listed in Supplementary Table S3 (available with the online version of this paper).

#### Cloning of the *comL* and *ssb* genes and overexpression of the recombinant proteins.

All DNA manipulations were performed according to standard techniques (Maniatis *et al.*, 1982). Full-length (FL) *comL* and *ssb* genes were amplified by PCR from MC58 genomic DNA using the primers listed in Supplementary Table S4 (available with the online version of this paper). The FL *comL* gene was cloned into the expression vector pET28b(+) (Novagen) with a C-terminal 6×His-tag, yielding plasmid pAVB1 (Supplementary Table S1). The vector pAVB1 was further used as template in the construction of partial N-terminal and C-terminal ComL constructs using the primers listed in Supplementary Table S4. The FL *ssb* gene was cloned into the vector pQE-30 (Qiagen) with an N-terminal 6×His-tag, yielding plasmid pEH1 (Supplementary Table S1). The recombinant proteins were overexpressed in *E. coli* ER2566 (New England Biolabs).

#### Purification of recombinant ComL and SSB proteins.

*E. coli* ER2566 cells overexpressing meningococcus ComL, SSB or ComL partial protein were grown in LB medium containing 50 µg kanamycin ml^−1^ at 37 °C with shaking. The cells were moved to 18 °C at OD_600_ 0.6, induced with 0.5 mM IPTG after 30 min and grown at 18 °C overnight. The cells were harvested by centrifugation at 4000 ***g*** for 20 min and frozen at −70 °C. The full-length ComL protein was purified from membrane-enriched fractions and solubilized in 1 % n-dodecyl β-maltoside (DDM) (Glycon). Specifically, the cell pellet was resuspended in phosphate buffer (50 mM NaH_2_PO_4_, 300 mM NaCl, pH 8.0) with the Complete protease inhibitor without EDTA (Roche Applied Science) and benzonase (Merck), and lysed by passing three times through a French press [103 500 kPa, (Thermo Electron)]. Unbroken cells were removed by centrifugation twice at 4000 ***g*** for 10 min and the membrane-enriched fraction was collected by ultracentrifugation (150 000 ***g***, 90 min). The membrane pellet was resuspended in a phosphate buffer (pH 8.0) containing 10 mM imidazole and 1 % DDM and left to solubilize overnight on a roller at 4 °C. Unsolubilized material was removed by ultracentrifugation (150 000 ***g***, 90 min). The supernatant was added to a Ni-NTA agarose column (Qiagen), washed and eluted with phosphate buffer (pH 8.0) containing 0.1 % DDM and increasing amounts of imidazole up to 250 mM. Fractions containing the purified recombinant protein were pooled and dialysed against buffer containing 50 mM NaH_2_PO_4_, pH 8.0, 300 mM NaCl and 0.1 % DDM. The proteins ComL_18–267_ and SSB were purified from the soluble fraction. In detail, the cell pellets were resuspended in phosphate buffer (50 mM NaH_2_PO_4_, 300 mM NaCl, pH 8.0) with the Complete protease inhibitor without EDTA (Roche Applied Science) and benzonase (Merck), and lysed by sonication. The lysates were cleared by centrifugation at 16 000 ***g*** for 40 min and the supernatant was passed through a Ni-NTA column, and washed and eluted with phosphate buffer (pH 8.0) containing increasing amounts of imidazole up to 250 mM. Fractions containing the purified recombinant proteins were pooled and dialysed against a buffer containing 50 mM NaH_2_PO_4_, pH 8.0, and 300 mM NaCl.

#### Solid-phase overlay assay (Far-Western analysis).

Protein–protein interactions between ComL, PilQ and PilQ partial proteins were assessed by a solid-phase overlay assay as described previously (Balasingham *et al.*, 2007). Briefly, 1 µg purified recombinant PilG, PilQ (Frye *et al.*, 2006), PilP, ComL, SSB proteins and BSA were separated by SDS-PAGE and transferred onto nitrocellulose membranes (Hybond-C Extra, Amersham GE Healthcare) in Towbin transfer buffer (25 mM Tris/HCl, 192 mM glycine, 20 % methanol, 0.1 % SDS, pH 8.3). The membranes were briefly washed twice with renaturing buffer (0.25 % gelatin, 0.5 % BSA, 0.2 % Triton X-100, 10 mM Tris/HCl, 5 mM β-mercaptoethanol, 100 mM NaCl, pH 7.5), and the proteins were renatured by incubation at 4 °C overnight in the same buffer. For the detection of protein–protein binding, the membranes were incubated for 3 h with 1 µg purified FL or partial proteins of ComL, PilG or PilQ in 10 ml renaturation buffer and washed in Tris-buffered saline (100 mM Tris/HCl, 150 mM NaCl, pH 7.5). Bound ComL, PilG or PilQ was detected with specific rabbit antisera. The PilQ–PilP interaction was used as a positive control (Balasingham *et al.*, 2007) and BSA was used as a negative control. The experiment was repeated at least three times.

#### Labelling of DNA substrates.

Oligonucleotides were end-labelled with [γ-^32^P]ATP (Perkin Elmer) using T4 polynucleotide kinase (New England Biolabs) as described by the manufacturer. Labelled oligonucleotides were separated from free nucleotides on 20 % non-denaturing PAGE gels and extracted by diffusion into water. Double-stranded labelled substrates were generated by mixing them with an equal molar amount of complementary unlabelled oligomer, heating them to 95 °C for 5 min and slow cooling to room temperature. The concentration of the double-stranded DNA substrate was estimated by dot quantification on agarose plates containing ethidium bromide as described elsewhere (Maniatis *et al.*, 1982), using unlabelled DNA of known concentration as the standard.

#### Electrophoretic mobility shift assay (EMSA).

For EMSAs, 4.5 fmol labelled DNA was mixed with 4 µl 2.5× gel shift buffer [25 % (w/v) glycerol, 12.5 mM MgCl_2_, 0.05 mg BSA ml^−1^, 2.5 mM DTT] and 5 µl protein diluted in 50 mM NaH_2_PO_4_, 300 mM NaCl, pH 8.0 and 0.1 % DDM in a final volume of 10 µl. The mixture was incubated at 37 °C for 15 min. Electrophoresis was carried out on 6 % polyacrylamide gels in Tris/glycine/EDTA buffer (Buratowski & Chodosh, 1996). The gels were dried, exposed to a phosphorImager cassette and scanned in a Typhoon scanner (both from Amersham GE Healthcare). The DNA substrates used in the assay are listed in Supplementary Table S2.

#### ComL rabbit immunization and antibody production.

Rabbit polyclonal antibodies were raised against the ComL_18–267_ protein as described previously (Tønjum *et al.*, 1995). Serum obtained 100 days after immunization was used for ComL detection. Anti-PilG, anti-PilP, anti-PilQ and anti-PilW sera were produced as described previously (Balasingham *et al.*, 2007; Frye *et al.*, 2006; Tønjum *et al.*, 1995).

#### Separation of outer and inner membranes by sucrose density gradient.

The meningococcus outer and inner membranes were separated by sucrose density gradient as described previously (Balasingham *et al.*, 2007). In brief, meningococcus M1080 cells were processed twice through a French press (103 500 kPa, Thermo Electron). Debris was removed by centrifugation at 10 000 ***g*** for 10 min. Sucrose gradient centrifugation was carried out in water with 3 mM EDTA, pH 8.0 (Masson & Holbein, 1983). The sample was transferred onto two layers of sucrose consisting of 3 ml 55 % (w/w) sucrose and 4 ml 15 % sucrose and centrifuged at 217 000 ***g*** and 4 °C for 5 h in an SW40Ti rotor (Beckman). The membrane fraction positioned at the interface was collected and diluted down to 30 % sucrose, applied to a discontinuous sucrose gradient consisting of 3 ml 50, 45, 40 and 35 % sucrose and centrifuged in an SW40Ti rotor at 180 000 ***g*** and 4 °C for 35 h. After fractionation, 10 µl samples were analysed by SDS-PAGE, followed by Coomassie blue staining and immunoblotting with the ComL-specific antibody.

## Results

### Search for DNA binding components in the inner membrane and soluble fractions

**Fig. 1.**
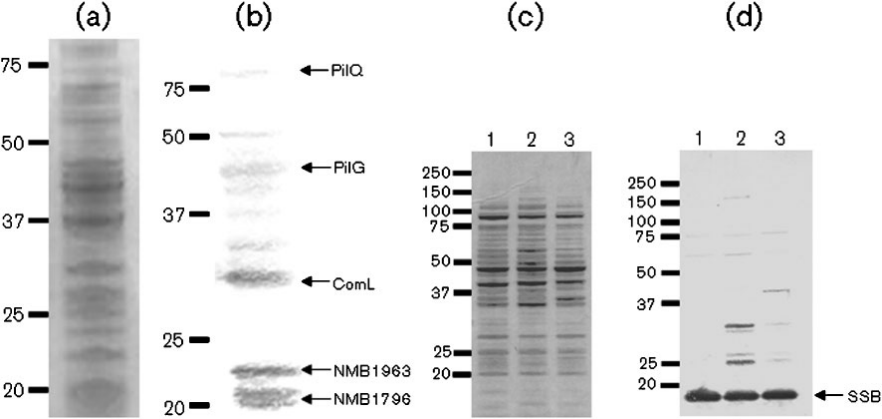
Identification of DNA binding components by a 1D solid-phase overlay approach. 1D Solid-phase overlay analysis of proteins isolated from the inner membrane and soluble fractions of neisserial strains. (a) 1D Coomassie-blue-stained SDS-PAGE of the inner membrane fraction of meningococcus strain H44/76. (b) Identification of DNA binding components in the inner membrane fraction of meningococcus strain H44/76. (c) 1D Coomassie-blue-stained SDS-PAGE of meningococcus soluble fractions. (d) Identification of SSB as a DNA binding component. Lanes: 1, H44/76; 2, MC58; 3, Z2491. The DNA substrate used in the assays depicted was ssDNA containing the 10 bp DUS. Positions of the size standards (kDa) are shown on the left and arrows on the right indicate the position of the proteins identified by MS analysis.

Proteins in the enriched meningococcus membranes were assessed with regard to their DNA binding activity in a solid-phase overlay assay. Among the multiple DNA binding bands detected in cellular fractions from meningococcus strains representing the major serogroups and one gonococcus strain, a 29 kDa band was the most predominant (Fig. 1a and b). This and four additional reproducibly detected bands were selected for identification. The experiment was performed with ssDNA and dsDNA substrates, with or without DUS. The 29 kDa DNA binding component was shown to interact with both ssDNA and dsDNA substrates, and the DNA binding observed was not enhanced by the presence of DUS.

In order to identify DNA binding proteins in the soluble fraction, chloroform extracts from all neisserial strains tested were subjected to a solid-phase overlay assay. This assessment yielded a predominant 19 kDa DNA binding component in all the isolates examined (Fig. 1c and d). The experiment was performed with ssDNA and dsDNA substrates, with or without DUS. The DNA binding component detected bound to ssDNA and dsDNA, without DUS specificity.

Gel slices containing the six predominant components that reproducibly bound DNA were excised from the parallel Coomassie-blue-stained gels (Fig. 1a and c) and submitted for peptide mass fingerprinting by MALDI-TOF-MS.

### Establishment of a 2D solid-phase overlay assay

**Fig. 2.**
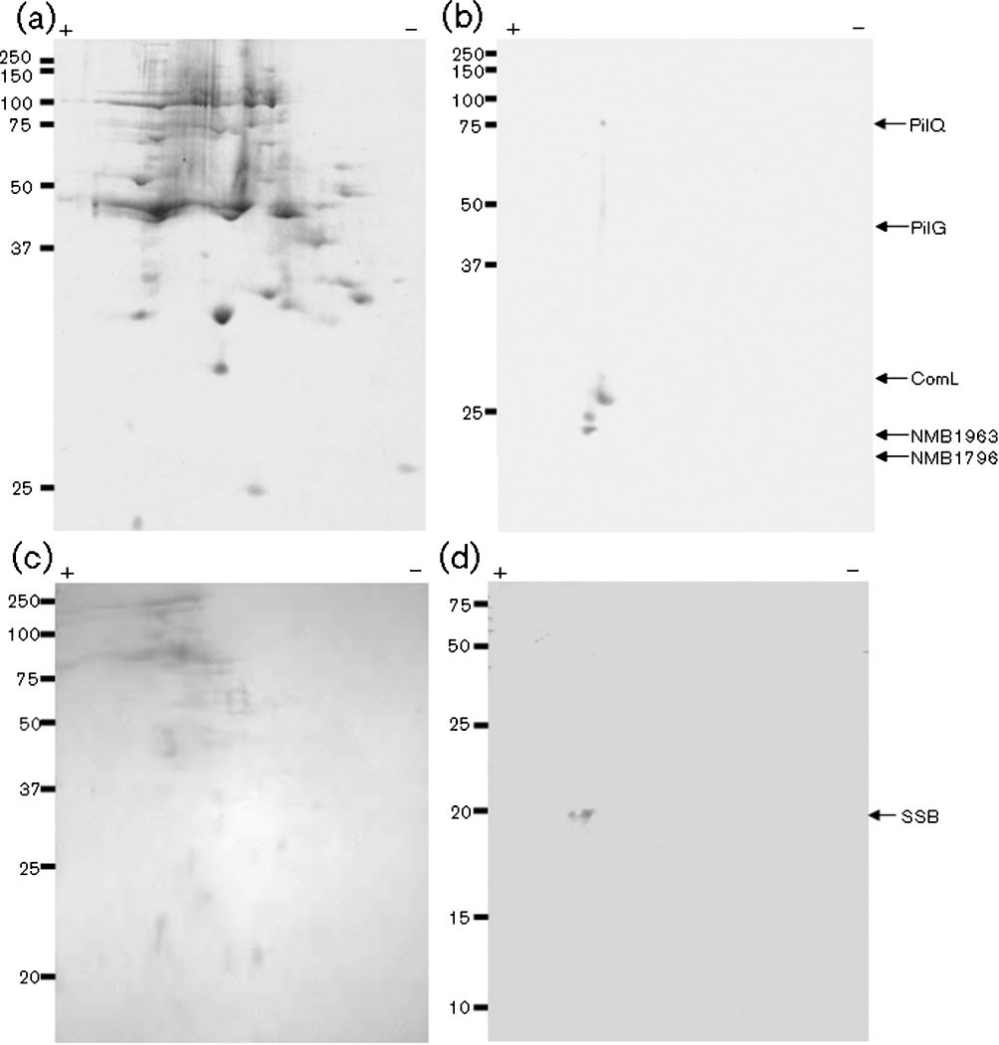
Identification of DNA binding components by a 2D solid-phase overlay approach and MS analysis. 2D Solid-phase overlay analysis of proteins isolated from the inner membrane and soluble fractions of neisserial strains. (a) 2D Coomassie-blue-stained gel of the inner membrane fraction of meningococcus strain MC58. (b) Identification of DNA binding activity in the inner membrane fraction of MC58 after 2D gel electrophoresis. (c) 2D Coomassie-blue-stained gel of the soluble fraction of MC58. (d) Identification of SSB DNA binding activity in the soluble fraction of MC58 by using the 2D approach. The DNA substrate used in the assays depicted was ssDNA substrate containing the 10 bp DUS. Positions of the size standards (kDa) are shown on the left and arrows on the right indicate the positions of the proteins identified by MS analysis.

In order to improve the resolution of protein bands observed in 1D SDS-PAGE, a 2D electrophoresis and subsequent solid-phase overlay assay was performed with the neisserial inner membrane and soluble fractions. Particularly for the inner membrane fraction, the 2D protein separation yielded a higher discrimination between reacting spots of inner membrane fractions in comparison with the 1D separation (Fig. 2). Gel slices containing the six predominant components that reproducibly bound DNA were excised from the parallel Coomassie-blue-stained gels (Fig. 2a and c) and analysed by using MS.

### Identification of the DNA binding components by peptide mass fingerprinting/MALDI-TOF-MS

The DNA binding proteins were identified by MALDI-TOF-MS analysis of trypsin-treatment-derived peptides from gel slices (Table 1). The predominant membrane-associated components that repeatedly exhibited DNA binding were identified as the competence lipoprotein ComL (47.9 % sequence coverage, 14 peptides assigned), the pilus biogenesis protein PilG (21.7 % sequence coverage, 11 peptides assigned), NMB1796, a predicted flavoprotein (36.3 % sequence coverage, six peptides assigned), and NMB1963, a periplasmic transport protein (35.7 % sequence coverage, eight peptides assigned). No recognized DNA binding motifs were found in the predicted amino acid sequences of either of these proteins. The additional identification of PilQ in this fraction (22.5 % sequence coverage, 15 peptides assigned) probably either represents contamination by abundant outer membrane proteins due to an incomplete membrane separation or is due to the fact that PilQ directly interacts with inner membrane proteins. PilQ and PilG have been previously found to bind DNA (Assalkhou *et al.*, 2007; Lång *et al.*, 2009).

**Table 1.** Characteristics of the DNA binding proteins identified by solid-phase overlay assay and MS analysis and phenotypic traits of the corresponding null mutants nv, Not viable; Wt, wild-type levels.

Protein identified	Predicted size (kDa)	Putative function	Colony morphology*	Extracellular pilus expression	Competence for transformation
**Inner membrane fraction**					
ComL NMB0703	29	Competence, peptidoglycan-related function	nv	nv	nv
Hypothetical protein NMB1796	20	Predicted flavoprotein	agg+	Wt	Competent
Hypothetical protein NMB1963	21	Periplasmic transport protein	agg+	Wt	Competent
PilG NMB0333	39	Pilus biogenesis	agg−	Absent	Non-competent (Tønjum *et al.*, 1995)
PilQ NMB1812	80	Pilus biogenesis	agg−	Absent	Non-competent (Frye *et al.*, 2006)
**Soluble fraction**					
SSB† NMB1460	19	ssDNA-binding protein	nv	nv	nv

* The colony morphology is described as agglutinating (agg+) and non-agglutinating (agg−).

† SSB was the single predominant component identified in the soluble fraction.

In the soluble fraction, the predominant 19 kDa DNA binding protein detected was identified as the meningococcus orthologue of the *E. coli* SSB (59.8 % sequence coverage, 10 peptides assigned). No DNA binding motif was identified in the sequence of the neisserial SSB orthologue by bioinformatic analyses, although ssDNA binding activity in the *E. coli* orthologue of SSB has previously been mapped to its C-terminal part, thus solving the crystal structure of the DNA binding domain (Savvides *et al.*, 2004).

### Phenotypes of meningococcus null mutants

Meningococcus null mutants corresponding to the DNA binding proteins identified were constructed when possible and examined with regard to their pilus-related phenotypes. The *comL* and *ssb* null mutants, however, were not viable, which corroborates the findings that ComL and SSB homologues are essential in other organisms. The *pilG* and *pilQ* null mutants, which are defective in pilus biogenesis, were non-competent for transformation as described previously (Tønjum *et al.*, 1995, 1998). The NMB1796 and the NMB1963 (Monaco *et al.*, 2006) null mutants were competent for transformation and exhibited wild-type levels of pilus expression (Table 1), suggesting that they are not involved in transformation and that their detection most likely represents a non-specific binding of DNA. Moreover, the identified ORFs encoding proteins that bind DNA were searched for the presence of DUS. DUS located inside ORFs has been shown to be biased towards DNA repair, recombination and replication genes (3R genes), thus indicating a potential role of transforming DNA in genome maintenance (Davidsen *et al.*, 2004). Nevertheless, none of the genes encoding the DNA binding proteins identified contained DUS within their coding sequence.

### ComL co-purifies with the meningococcus outer membrane

**Fig. 3.**
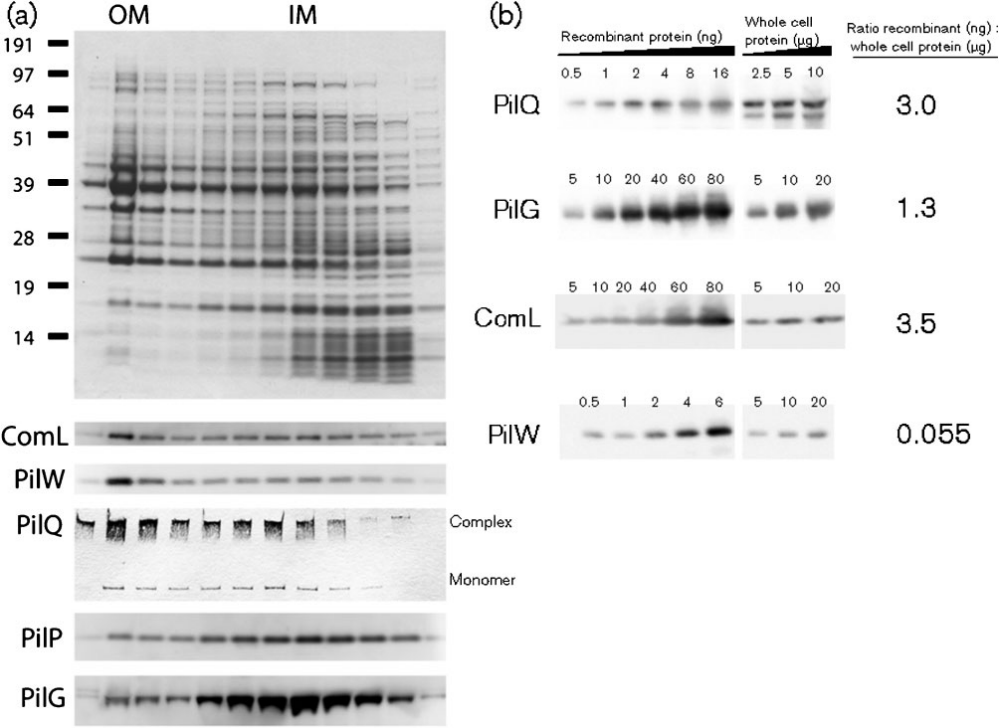
ComL co-purifies with the outer membrane and is expressed at high levels. (a) Analysis of cellular fractions after separation of outer and inner membranes from *N. meningitidis* M1080 by sucrose-gradient centrifugation. Samples from each fraction were separated by SDS-PAGE and stained with Coomassie blue (top panel) or detected by immunoblotting using antibodies against ComL, PilG, PilP, PilQ and PilW (lower panels). (b) Quantitative immunoblotting of a defined amount of *N. meningitidis* M1080 cellular suspension using the same antibodies as in (a).

In order to determine the subcellular localization of ComL, meningococcus outer and inner membranes were separated by sucrose density gradient centrifugation. Fractions from the sucrose gradient were analysed by immunoblotting using antisera against ComL, PilG, PilP, PilQ and PilW (Fig. 3a). PilQ and PilP have previously been shown to reside in the outer and inner membrane, respectively (Balasingham *et al.*, 2007), while PilW resides in the outer membrane, interacting with PilQ (Carbonnelle *et al.*, 2005; Trindade *et al.*, 2008). The main amounts of ComL and PilW peaked with PilQ in the higher-density outer membrane fractions, while PilG and the lipoprotein PilP were concentrated in the inner membrane gradient fractions with very low levels detected in the higher-density fractions, demonstrating that ComL co-purifies with the outer membrane. ComL was shown to be expressed at levels as high as the secretin PilQ (Fig. 3b).

### DNA binding properties of recombinant meningococcus ComL and SSB

**Fig. 4.**
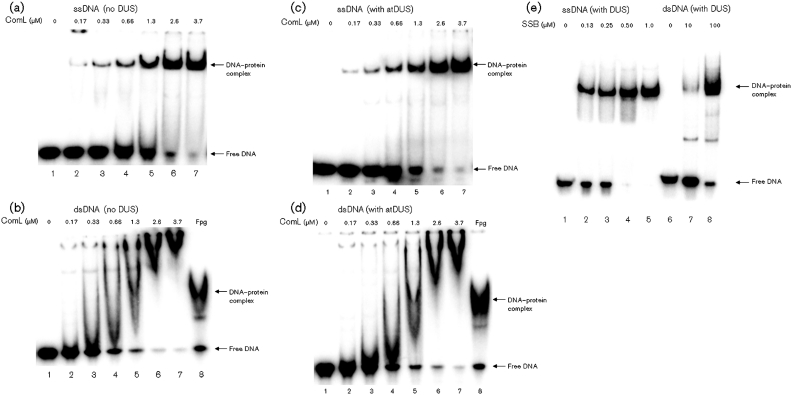
*N. meningitidis* recombinant ComL and SSB exhibit DNA binding activity. The DNA binding abilities of purified recombinant full-length ComL and SSB proteins were assessed by EMSA. (a) ComL binds to ssDNA substrate without the DUS. A protein concentration between 0.66 and 1.3 µM is required to shift 50 % of the ssDNA substrate, indicating an apparent dissociation constant of 0.66–1.3 µM. (b) ComL binds to dsDNA substrate without DUS. A protein concentration between 0.33 and 0.66 µM is required to shift 50 % of the dsDNA substrate, indicating an apparent dissociation constant of 0.33–0.66 µM. A 50 ng sample of recombinant meningococcus Fpg was used as a positive control. (c, d) The same results were obtained with ssDNA and dsDNA substrates with the 12 bp DUS. (e) SSB binds to both ssDNA and dsDNA, though with a strong preference for ssDNA. A protein concentration between 0.25 and 0.5 µM is required to shift 50 % of the ssDNA substrate, and between 10 and 100 µM is required to shift 50 % of the dsDNA substrate. This indicates an approximately 150-fold higher affinity for ssDNA than for dsDNA. atDUS, 12 bp DUS.

**Fig. 5.**
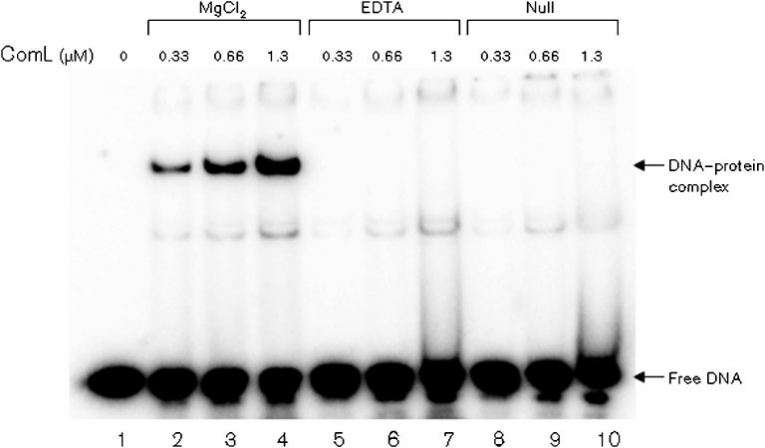
*N. meningitidis* recombinant ComL DNA binding activity is Mg^2+^-dependent. A panel of EMSA buffers was used to assess the DNA binding activity of the purified full-length ComL protein. A protein–DNA complex shift was observed only with buffer containing MgCl_2_. This shift was abolished when buffers containing EDTA or no additives were used.

Native ComL and SSB were identified as neisserial DNA binding proteins by using 1D and 2D solid-phase overlay assays in combination with MS analysis (Figs 1 and 2) (Lång *et al.*, 2009). The DNA binding activity of recombinant ComL and SSB was verified by EMSA (Fig. 4). The ComL DNA binding activity observed was dependent on the presence of Mg^2+^ and was abolished when EMSA buffers containing EDTA or no additive were used (Fig. 5). Similar results were obtained with all DNA substrates employed (Supplementary Table S2). The DNA binding activities of recombinant ComL and SSB were not DUS-specific (Fig. 4).

### ComL and SSB predicted structures

**Fig. 6.**
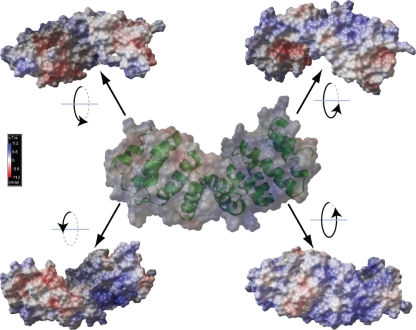
*N. meningitidis* ComL 3D model. The *N. meningitidis* MC58 ComL hypothetical 3D structure generated by the phyre threading server is shown in cartoon form (centre) with the concurrent surface representation overlaid. The charge distribution, calculated using APBS, is shown on the surface (blue = positive, red = negative). The ComL surface is shown in four further images (in corners) rotated progressively around a horizontal axis in order to give a complete overview of the charge distribution. The large areas of positive charge may indicate that they play a role in DNA binding.

Predicted 3D models for the structure of ComL and SSB were generated using the phyre service (Bennett-Lovsey *et al.*, 2008). The sequence identity of ComL relative to known structures was below the 30 % level required to generate a confident homology model, with the best results returning between 11 and 18 % sequence identity. However, the e-values of these alignments were very low (<1e^−19^), which allowed for the generation of a hypothetical structure by threading. The most compatible structures returned by the server were characterized due to the presence of a tetratricopeptide repeat (TPR) structure; these findings included *Pseudomonas aeruginosa* PilF (Kim *et al.*, 2006), which was also the component closest in length to ComL. Since sequence-based DNA binding predictions with regard to ComL were uniformly negative, the threading model structure was used as a basis to investigate the possible distribution of positive charges on the surface, thereby yielding a possible explanation for the DNA binding observed. A 3D model structure was generated and the charge on the molecular surface was calculated by using the Adaptive Poisson–Boltzmann Solver (Fig. 6) (Baker *et al.*, 2001). Charge distribution on the ComL surface is shown for a representative range of orientations, demonstrating that there are several regions of positive charge (blue), which may function in specific or non-specific DNA binding.

**Fig. 7.**
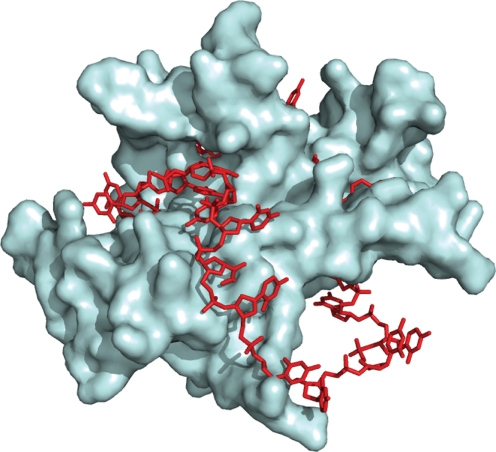
*N. meningitidis* SSB 3D model. The *N. meningitidis* MC58 SSB predicted 3D structure modelled by homology using the swiss-model service is shown here as a dimer, substituted into the crystal structure for *E. coli* SSB bound to ssDNA. This illustrates a functional mode for ssDNA binding by SSB.

In contrast, neisserial SSB had 33.6 % sequence identity with a known 3D crystal structure from *Thermus aquaticus* (Fedorov *et al.*, 2006), which allowed the automated generation of a homology model by means of the swiss-model server pipeline (Arnold *et al.*, 2006). An additional structural SSB homologue from *E. coli* has been crystallized in its multimeric form, bound to ssDNA (Raghunathan *et al.*, 2000). The 3D model structure for neisserial SSB has been substituted as a dimer into this structure in order to illustrate the predicted meningococcus SSB mode of binding for ssDNA (Fig. 7).

### ComL directly interacts with N-terminal PilQ

**Fig. 8.**
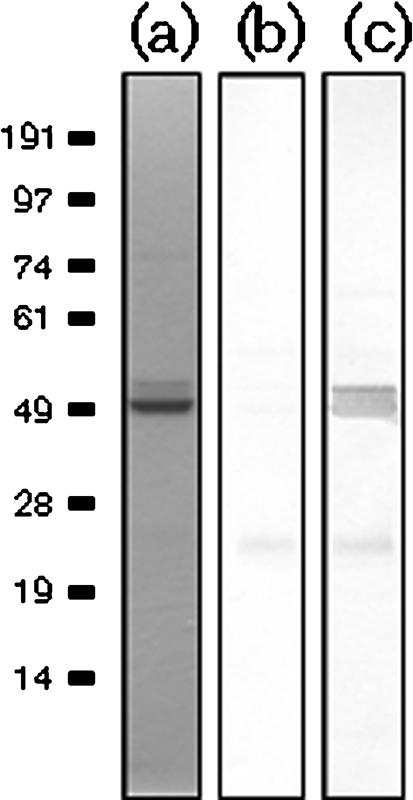
The lipoprotein ComL directly interacts with PilQ. Solid-phase overlay interaction study between ComL_18–267_ and PilQ_25–354_ on the blot and overlay with ComL_18–267_, and detection with ComL antibody. (a) Coomassie blue staining of PilQ_25–354_. (b) Negative control with PilQ_25–354_ protein on the membrane probed with ComL antibody alone. (c) PilQ_25–354_ protein on the membrane, probed with ComL antibody after an overlay of ComL_18–267_. A stronger staining for PilQ_25–354_ and its degradation product is seen when an overlay of ComL_18–267_ is added before detection with antibody, indicating an interaction between PilQ_25–354_ and ComL_18–267_.

A solid-phase overlay assay in the form of a Far-Western analysis was employed to determine if there was interaction between the ComL and PilQ proteins (Fig. 8). This revealed that the ComL and N-terminal PilQ proteins directly interact *in vitro*.

## Discussion

In this study, we searched cellular fractions from a representative panel of neisserial strains for DNA binding components, using 1D and 2D electrophoresis combined with a solid-phase overlay approach. The DNA binding proteins were identified by MS analysis. Enrichment of the membrane and the soluble fractions were important steps contributing to the generation of target solutions with reduced complexity, as compared with meningococcus whole-cell extracts, for the identification of DNA binding proteins. The 2D solid-phase overlay strategy further increased the spatial resolution of the proteins in comparison with the 1D approach. Employing peptide mass fingerprinting by MALDI-TOF-MS enabled protein identification from small amounts present in the 1D/2D gel spots which exhibited DNA binding.

The predominant membrane components exhibiting DNA binding activity were identified as ComL and PilG, as previously reported (Lång *et al.*, 2009). In this context, we wanted to characterize ComL further. In EMSA, ComL was shown to bind DNA in a Mg^2+^-dependent manner, indicating that Mg^2+^ facilitates the direct binding of DNA (Fig. 5). A hypothetical 3D structure for ComL was generated and the charge distribution on the ComL molecular surface was predicted, suggesting that there are several regions of positive charge, which may function as specific or non-specific DNA binding regions. ComL was also shown to have structural homology with the *P. aeruginosa* pilus biogenesis protein PilF (Kim *et al.*, 2006), which is homologous to the neisserial outer membrane lipoprotein PilW engaged in pilus biogenesis (Carbonnelle *et al.*, 2005; Trindade *et al.*, 2008).

The neisserial ComL protein has been suggested to contribute to DNA uptake by cleavage of the peptidoglycan layer during transformation (Fussenegger *et al.*, 1996, 1997). The gonococcal *comL* gene exists in a single copy, which is transcribed in the opposite direction to the neighbouring *comA* gene and encodes a periplasmic lipoprotein with a relatively high theoretical pI (9.03). The *comL* and *comA* gene pair has a common DUS-containing transcriptional terminator in an appropriate position for joint use (Jose *et al.*, 2003). The ComL orthologue in *E. coli*, YfiO, is anchored to the outer membrane and is a member of the β-barrel assembly machinery (the BAM complex) (Wu *et al.*, 2005). Recently, ComL was referred to as an orthologue of BamD, which suggests that neisserial ComL is associated with the outer membrane and is involved in outer membrane protein biogenesis (Knowles *et al.*, 2009). This notion was supported by our membrane separation (Fig. 3a). Orthologues of ComL are conserved among both neisserial and other Gram-negative bacterial species (Fussenegger *et al.*, 1996; Malinverni *et al.*, 2006). Bioinformatic inferences suggest that the *comL* gene encodes TPRs, and that the resulting structure presents several large regions of positive charge, which may act as either ssDNA or dsDNA binding sites. Still, it is not possible to determine from these structural predictions whether the apparent DNA binding capacity is related to the TPRs. TPRs have been predicted for neisserial PilW and *Pseudomonas* PilF, and have been suggested to serve a functional role in PilQ stabilization (Koo *et al.*, 2008; Trindade *et al.*, 2008). Thus, as previously suggested, the TPRs predicted could be of functional importance in mediating protein–protein interactions between ComL and other BAM proteins (D’Andrea & Regan, 2003; Knowles *et al.*, 2009). No potential DNA–protein interactions have been reported for TPR-containing proteins (D’Andrea & Regan, 2003), though the predicted positively charged regions of ComL may interact with DNA in a non-specific manner. Site-directed point mutations will enable the elucidation of the potential role of TPRs in ComL-mediated DNA binding or protein–protein interactions.

A significant decrease in the transformation rate of a gonococcal *comL* mutant has been documented, suggesting a role of the lipoprotein ComL in the neisserial transformation process in interaction with the peptidoglycan layer (Fussenegger *et al.*, 1996). Moreover, neisserial ComL has been suggested to be involved in the folding of outer membrane proteins (Knowles *et al.*, 2009). The true function of ComL is difficult to assess since most mutants of this essential component are lethal. It is of note that Fussenegger and co-workers managed to generate a viable gonococcal *comL* mutant, expressing a truncated version of the protein, therefore indicating the importance of an intact N terminus in ComL protein expression and function (Fussenegger *et al.*, 1996). Meningococcal *comL* null mutants were also non-viable, indicating that ComL is essential in neisserial species (Table 1). The *pilQ* and *pilG* null mutants, defective in pilus biogenesis, were non-competent for transformation. For these two components, the biological significance of their DNA binding capabilities is complicated by the fact that they participate in type IV pilus biogenesis, which in turn is required for competence. Thus, it is a conundrum as to whether the lack of competence in these mutants is due to a defect in their direct binding of DNA or whether this lack is indirect through pilus biogenesis.

SSB, the predominant DNA binding protein detected in the soluble neisserial cell fractions, is involved in processing ssDNA intermediates during DNA replication, repair and recombination in *E. coli* (Shereda *et al.*, 2008). Furthermore, SSB proteins are conserved, serve critical functions in genome maintenance and are indispensable for cell survival among both prokaryotes and eukaryotes (Fanning *et al.*, 2006; Shereda *et al.*, 2008; Zou *et al.*, 2006). In our hands, neisserial SSB was found to bind both ssDNA and dsDNA, which is consistent with findings on SSB in other prokaryotes, in which SSB either binds to ssDNA or intercalates itself into dsDNA, thereby disrupting it (Makhov & Griffith, 2006; Makhov *et al.*, 2009; Mapelli *et al.*, 2005). Previously, the genome of *Bacillus subtilis* was shown to comprise two paralogous SSB genes, *ssb* and *ywpH*. Interestingly, the proteins encoded by *ssb* and *ywpH* have distinctive expression patterns, with SSB being essential for cell survival, while YwpH is required for natural transformation (Lindner *et al.*, 2004). In the *ywpH* null mutant, the transformation rate was reduced fivefold, whereas the *ssb* null mutant was not viable (Lindner *et al.*, 2004; Ogura *et al.*, 2002). In *Haemophilus influenzae* RD KW20, gene expression analyses have revealed that the SSB orthologue HI0250 is induced 3.4-fold during the competent state (Redfield *et al.*, 2005). Based on the broad conservation of SSB functions and the documented role of SSB in transformation, we suggest that neisserial SSB might also have a functional role in transformation. As in *E. coli* (Shereda *et al.*, 2008), meningococcus *ssb* null mutants were non-viable, and meningococcus SSB phenotypes directly associated with transformation could therefore not be assessed (Table 1).

The purpose of this study was to identify and characterize neisserial DNA binding proteins and assess their potential relevance for transformation, putting a special emphasis on the characterization of ComL and SSB and their DNA binding properties. The neisserial DUS has been shown to mediate selective uptake of DNA through transformation; thus the search for DUS-specific DNA binding components was a priority. However, this endeavour turned out to be a difficult task since none of the DNA binding proteins identified bound DNA in a DUS-specific manner, nor contained the DUS sequence within their ORFs. The difficulties and challenges in identifying a potential DUS-specific binding protein are multiple, since functional and technical obstacles obscure the hunt for an unknown or putative component. If it does exist, the cellular location of DUS selectivity is not yet known. This search for DNA binding candidates targeted the inner membrane and cytoplasm, and a DUS-specific protein in these fractions and the neisserial outer membrane has not yet been identified. This negative search result could well be due to technical limitations, in that the *in vitro* conditions in the solid-phase overlay approach employed do not reflect the DNA binding that goes on *in vivo*. The solid-phase overlay assay also has limitations in the form of the level of protein expression, protein folding and renaturation abilities after SDS-PAGE. Thereby, not all DNA binding candidates will be detected using this method, and DUS-specific DNA binding might be of such a subtle and transient nature that it is not detected in this assay. Yet, the reproducible identification of PilG, ComL and PilQ DNA binding by this approach (Lång *et al.*, 2009), in addition to other independent assays (Assalkhou *et al.*, 2007), is strong evidence that the method is indeed valid for detecting a number of DNA binding proteins in general.

This study strengthens previous findings on potential direct roles for ComL, PilG and PilQ in transformation. Additional studies are warranted to provide new insights into the functional relationships between these and other proteins involved in the transformation process. Characterization of the physical interactions between ComL and SSB with DNA, in addition to other proteins, will contribute to a better understanding of how transforming DNA is processed in meningococcus cells and will further elucidate the neisserial DNA uptake and genome integration process.

## Supplementary Material

Supplementary table
