# Supplementary material for: Structure–function relationships of the competence lipoprotein ComL and SSB in meningococcal transformation
Source: Microbiology (Reading). 2011 May;157(Pt 5):1329–42. doi: 10.1099/mic.0.046896-0 (PMC3140584; doi:10.1099/mic.0.046896-0)
Supplement: Supplementary table [file supp_157_5_1329__index.html]

Structure–function relationships of the competence lipoprotein ComL and SSB in meningococcal transformation — Supplementary Tables 

# Structure–function relationships of the competence lipoprotein ComL and SSB in meningococcal transformation

## Supplementary tables

### Structure–function relationships of the competence lipoprotein ComL and SSB in meningococcal transformation, by A. V. Benam, E. Lång, K. Alfsnes, B. Fleckenstein, A. D. Rowe, E. Hovland, O. H. Ambur, S. A. Frye and T. Tønjum

*Microbiology* vol. **157**, part 5, pp. 1329 - 1342

**Supplementary Table S1.** Bacterial strains and plasmids employed in this study.   
**Supplementary Table S2.** DNA substrates employed in this study.   
**Supplementary Table S3.** Primers employed in construction of mutants.   
**Supplementary Table S4.** Primers employed in ComL and SSB recombinant protein construction.   
All tables are available as a single PDF (177 KB).
