# Supplementary material for: Structure–function relationships of the competence lipoprotein ComL and SSB in meningococcal transformation
Source: Microbiology (Reading). 2011 May;157(Pt 5):1329–42. doi: 10.1099/mic.0.046896-0 (PMC3140584; doi:10.1099/mic.0.046896-0)
Supplement: Supplementary table [file supp_157_5_1329__1.pdf]

Supplementary Table S1. Bacterial strains and plasmids employed in this study

Strain or plasmid	Relevant characteristic	Reference or source
***N. meningitidis* strains**		
Z2491	Serogroup A, isolated in Gambia 1983	Crowe *et al.* (1989)
MC58	Serogroup B, isolated in the UK 1983	McGuinness *et al.* (1991)
H44/76	Serogroup B, isolated in Norway 1976	Holten (1979)
M1080	Serogroup B, isolated in the USA, 1969	Frasch & Chapman (1972)
8013	Serogroup C	Caugant *et al.* (1986)
H44/76 ΔPilG	*pilG*::*m*Tn*Erm* transposon insertion	Tønjum *et al.* (1995)
H44/76 ΔPilQ	H44/76 strain with mTn::cm#21 in *pilQ*	Tønjum *et al.* (1998)
***N. gonorrhoeae* strain**		
N400	MS11 subclone, *recA6*(*tetM*)*	Tønjum *et al.* (1995)
***E. coli* strains**		
ER2566	Expression strain with a chromosomal copy of the T7 RNA polymerase gene	New England Biolabs
XL-1 Blue	*recA1 endA1 gyrA96 thi*-1 *hsdR17 supE44 relA1 lac* [F′ *proAB lacI*^{q}ZΔM15 Tn*10*Tet^{r}]	Stratagene
**Vectors**		
pBSK+	General cloning vector, Amp^{r}	Stratagene
pET28b(+)	Expression vector based on a T7-promoter-driven system, His tag, Kan^{r}	Novagen
pQE-30	Expression vector based on a T5-promoter-driven system, His tag, Amp^{r}	Qiagen
pAVB1	pET28b harbouring *comL* from MC58	This study
pHH10	pET28b harbouring $ComL_{18–267}$ insert	This study
pHH11	pET28b harbouring $ComL_{18–140}$ insert	This study
pHH12	pET28b harbouring $ComL_{139–267}$ insert	This study
pEH1	pEQ-30 harbouring *ssb* from MC58	This study

**recA6* is an IPTG-inducible allele of *recA*.

Benam, A. V., Lång, E., Alfsnes, K., Fleckenstein, B., Rowe, A. D., Hovland, E., Ambur, O. H., Frye, S. A. & Tønjum, T. (2011). Structure–function relationships of the competence lipoprotein ComL and SSB in meningococcal DNA transformation. *Microbiology* **157**, 1329–1342.

Supplementary Table S2. DNA substrates employed in this study

Substrate	Sequence (5′→3′)*	DUS length
**ssDNA**		
T_1	CAACAACAACAACA**GCCGTCTGAA**CCAAATTCAGACGGCAACAACAACAACA	+, 10 bp
T_3	CAACAACAACAACAGGCCTGTCATCCAAACTGACAGGCCAACAACAACAACA	–
HH7†	AACAACAACAA**ATGCCGTCTGAA**CCAACATGCCGTCTGAAAACAACAACAAC	+, 12 bp
HH9	GTTGTTGTTGTT**ATGCCGTCTGAA**GTTGGATGCCGTCTGAATTGTTGTTGTT	+, 12 bp
HH10†	AACAACAACAAAAGGCCTGTCATCCAACTTCCGGACAGTAAACAACAACAAC	–
**dsDNA**		
T_1T_2	CAACAACAACAACA**GCCGTCTGAA**CCAAATTCAGACGGCAACAACAACAACA	+, 10 bp
	TGTTGTTGTTGTTGCCGTCTGAATTTGG**TTCAGACGGC**TGTTGTTGTTGTTG	
HH7, HH8†	AACAACAACAA**ATGCCGTCTGAA**CCAACATGCCGTCTGAAAACAACAACAAC	+, 12 bp
	GTTGTTGTTGTTTTCAGACGGCATGTTGG**TTCAGACGGCAT**TTGTTGTTGTT	
HH10, HH11†	AACAACAACAAAAGGCCTGTCATCCAACTTCCGGACAGTAAACAACAACAAC	–
	GTTGTTGTTGTTTACTGTCCGGAAGTTGGATGACAGGCCTTTTGTTGTTGTT	

*The DNA uptake sequence (DUS) is indicated by bold type.

†DNA substrates used in electromobility shift analysis.

Supplementary Table S3. Primers employed in construction of mutants

Primer	Sequence (5′→3′)*	Restriction sites
	***comL* (NMB0703)**	
KH45	GCGCTAGCTCTTGGGTAATCTGGGCATC	*Nhe*I
KH46	GCGGATCCGTTTGTTGACGACGATGACG	*Bam*HI
KH47	GCGAATTCACGAGCTGAACAGCAGCAAT	*Eco*RI
KH48	GCAAGCTTCAATATGGCGAGCGATTCTT	*Hin*dIII
	**NMB1796**	
KH57	GCGCTAGCAGGCTGCCCACCAAAATACT	*Nhe*I
KH58	GCGGATCCCGTTTACCGTACCGTTGCTT	*Bam*HI
KH59	GCGAATTCTCGTTGAAATCGGACATCTG	*Eco*RI
KH60	GCAAGCTTGCTATAAACACGCCGGTCAT	*Hin*dIII
	**NMB1963**	
KH61	GCGCTAGCTCAAAATACCGATGCCCAAT	*Nhe*I
KH62	GCGGATCCGCGGTTCGGACAAAACTTAC	*Bam*HI
KH63	GCGAATTCGGTAAGCCAAATCCGTCAAA	*Eco*RI
KH64	GCAAGCTTTTGCTCGGAAAACCCATAC	*Hin*dIII
	***ssb* (NMB1460)**	
KH73	GCGCTAGCGCCGATGAGGATGACTTTGT	*Nhe*I
KH74	GCGGATCCGCCGTCTATGGTTTCCAAAA	*Bam*HI
KH75	GCGAATTCATCCCGAAGTGCGCTATATG	*Eco*RI
KH76	GCAAGCTTGCCCCGTAAGGTTTAAGGTT	*Hin*dIII

*Restriction sites are underlined.

Supplementary Table S4. Primers employed in ComL and SSB recombinant protein construction

Primer	Sequence (5′→3′)*	Restriction sites
SAF72	CGTCTAGAAATAATTTTGTTTAACTTTAAGAAGGAGATATACCATGAAAAA AATTCTTTTAACGGTTT	*Xba*I
SAF73	CGCTCGAGGTGCCAGTAACGCCACCAGGGCATAT	*Xho*I
HH12	CGCCATGGCCACTCAAGGTACGGTCGATAA	*Nco*I
HH13	CGCTCGAGCGGGTCGCGGTCGGACCAGTCTT	*Xho*I
HH14	CGCCATGGACCCGAAAGCCAACCGCGAAGCGTAT	*Nco*I
EH001	TGGGATCCATGTCATTGAACAAAGTCATCCTCAT	*Bam*HI
EH002	CGAAGCTTTCAGAACGGGATATCGTCGTCAATGT	*Hin*dIII

*Restriction sites are underlined.

Benam, A. V., Lång, E., Alfsnes, K., Fleckenstein, B., Rowe, A. D., Hovland, E., Ambur, O. H., Frye, S. A. & Tønjum, T. (2011). Structure–function relationships of the competence lipoprotein ComL and SSB in meningococcal DNA transformation. *Microbiology* **157**, 1329–1342.

Supplementary References

Caugant, D. A., Frøholm, L. O., Bøvre, K., Holten, E., Frasch, C. E., Mocca, L. F., Zollinger, W. D. & Selander, R. K. (1986). Intercontinental spread of a genetically distinctive complex of clones of *Neisseria meningitidis* causing epidemic disease. *Proc Natl Acad Sci U S A* **83**, 4927–4931. CrossRef Medline

Crowe, B. A., Wall, R. A., Kusecek, B., Neumann, B., Olyhoek, T., Abdillahi, H., Hassan-King, M., Greenwood, B. M., Poolman, J. T. & Achtman, M. (1989). Clonal and variable properties of *Neisseria meningitidis* isolated from cases and carriers during and after an epidemic in The Gambia, West Africa. *J Infect Dis* **159**, 686–700. Medline

Frasch, C. E. & Chapman, S. S. (1972). Classification of *Neisseria meningitidis* group B into distinct serotypes. I. Serological typing by a microbactericidal method. *Infect Immun* **5**, 98–102. Medline

Holten, E. (1979). Serotypes of *Neisseria meningitidis* isolated from patients in Norway during the first six months of 1978. *J Clin Microbiol* **9**, 186–188. Medline

McGuinness, B. T., Clarke, I. N., Lambden, P. R., Barlow, A. K., Poolman, J. T., Jones, D. M. & Heckels, J. E. (1991). Point mutation in meningococcal por A gene associated with increased endemic disease. *Lancet* **337**, 514–517. CrossRef Medline

Tønjum, T., Freitag, N. E., Namork, E. & Koomey, M. (1995). Identification and characterization of *pilG*, a highly conserved pilus-assembly gene in pathogenic Neisseria. *Mol Microbiol* **16**, 451–464. CrossRef Medline

Tønjum, T., Caugant, D. A., Dunham, S. A. & Koomey, M. (1998). Structure and function of repetitive sequence elements associated with a highly polymorphic domain of the *Neisseria meningitidis* PilQ protein. *Mol Microbiol* **29**, 111–124. CrossRef Medline
